# Support of intelligent emergent materials to combat COVID-19 pandemic

**DOI:** 10.1007/s42247-021-00189-3

**Published:** 2021-03-10

**Authors:** Huseyin C. Yalcin, Ajeet Kaushik

**Affiliations:** 1 Qatar University Biomedical Research Center, Doha, Qatar; 2 NanoBioTech Laboratory, Health Systems Engineering, Department of Natural Sciences, Division of Sciences, Art, & Mathematics, Florida Polytechnic University, Lakeland, FL 33805 USA

The COVID-19 pandemic, associated with SARS-CoV-2 infection transmitted via human to human and cause life-threatening respiratory diseases, has emerged as an ever-increasing global health and economic crisis since its declaration by the World Health Organization (WHO) in early Jan 2020. Despite the development of several vaccines and initiation of vaccination programs, it is very likely that we will have to continue our lives under now became normal preventative measures for several more years. While this global battle against the pandemic is carried out on the frontlines by healthcare providers, another major effort are underway by scientists and engineers in research labs around the globe for investigating better therapies, detection systems, and safety aspects. In this unprecedented scenario, experts are seeking fast, practical, and effective ways to support healthcare providers in treating patients and prevent or slow further spread of the virus. In this dazzling race against time, materials science is one of the fields that is contributing significantly, due to a substantial cumulative knowledge that can be translated rapidly to clinical practice. Novel material approaches of tunable performance can be useful for various multi-tasking applications such as accurate diagnosis of viral infection from patient samples, sanitizing or preventing viral accumulation on surfaces, alternative sources and sanitation for personal protective equipment, effective delivery and binding of antiviral agents to the virus, reprogramming of the immune system, and even development of injectable synthetic compounds to compete with the virus in binding to viral receptors.

Such new knowledge needs to be communicated effectively with other talented and courageous minds to win this battle. With the abovementioned into consideration, *Emergent Materials,* a Springer Journal, has organized a special issue on exploring aspects of novel material science applications to manage COVID-19 pandemic effectively, and this timely issue is entitled as “Materials Science in the Battle against COVID-19”.

For this projected COVID-19 Special issue, we aimed to create a representative collection of reviews and original papers that would cover and highlight major relevant themes in the field of Materials Science, such as nanomedicine/nanoparticles, bio-sensors, personnel protective equipment, additive manufacturing, medical devices, and biomaterials/tissue engineering. We are glad to present a total of 22 high-quality articles from renowned research groups around the globe. We believe these high-quality peer-reviewed articles will introduce journal readers with urgently required emerging approaches needed for developing novel functional materials strategies to fight with COVID-19 pandemic with the guaranty of success.

The issue includes several perspective/review papers explaining potential impact of material science field for advancement of therapies, diagnotics, and preventative strategies from different perspectives. *Edirisinghe and co-workers* explain the importance of incorporation of novel biomaterials within “smart” face masks for prevention of transmission and enhancement of antiviral activity. Authors also emphasized environmentally acceptable material selection to minimize long-lasting effect on the environment. *Bencherif and co-workers* elegantly summarized emerging approaches used in the development of new therapies, such as virus deactivating surface coatings, biomaterials used for 2D/3D cell culture as drug screening models, organ on a chip technologies, and biomaterials for targeted antiviral drug delivery systems. While *Mozafari and co-workers* presented a general overview for the use of different nano-enabled biomaterial based approaches for addressing current pandemic as well as future pandemics, *Ashammakhi and co-workers* explained more specific applications such as tissue engineering/stem cell technologies, organoids and organ on a chip systems for advancing therapies, drug delivery approaches, and vaccines against SARS-COV-2. *Dubey and co-workers* focused on different biomaterials for the development of PPE kits such as protecting suits, gloves, masks, etc. as well as disinfection of the surfaces/surroundings.

Nanomedicine is one of the most promising fields to advance therapy of this infection, prescribed therapy assessment, and diagnostics of the disease. Special issue has several papers on relevant nanotechnology approaches. Being an active researcher as well as a clinician, *Chakravarthy and co-workers* elegantly summarized how the medical field would benefit from nanotechnology for the treatment and diagnosis of COVID-19 pandemic. *Tharayil and co-workers* presented different nanoparticle types for fast detection of SAR-COV-2 whereas *Kumar and co-workers* explained a variety of advanced nanoparticles for the treatment of COVID-19.

The recent SARS-COV-2 outbreak once again reminded us the importance of rapid and direct detection of respiratory disease viruses. Conventional methods for virus detection such as PCR tests are based on techniques relying on cell culture, antigen-antibody interactions, and nucleic acids, which require trained personnel as well as expensive equipment. Microfluidic technologies, on the other hand, can accurately detect respiratory tract viruses with high specificity. We included several papers in the special issue explaining novel biosensor technologies for enhanced diagnostics of SARS-COV-2. *Tekin and co-workers* summarized a variety of microfluidic-based virus detection methods for respiratory diseases and explained potential impacts of these techniques for the current pandemic by comparison with conventional diagnostic tools currently in use. *Inci and co-workers* elegantly explained different nanomaterials being used in diagnostic microfluidic platforms currently being developed for COVID-19. *Trabzon and co-workers* explained high affinity biosensors developed with quantum dots and how this novel technology can be applied for SARS-COV-2 detection in microfluidic systems. *Yuce and co-workers* presented different viral sensing approaches in microfluidic systems such as plasmonic biosensors, electrochemical biosensors, and magnetic biosensors focusing on application to SARS-COV-2 protein detection. *Jeerapan and co-*workers specifically explained in detail about the electrochemical biosensing/biolelectronics for microfluidic platforms for viral diagnostics.

Special issue contained several papers on development of functional biomaterials for SARS-COV-2. Antimicrobial surfaces can potentially prevent spread of viral infections. *Nastruzzi and co-workers* elegantly presented a new biomaterial, which the authors named as gold hard anodized (GHA) materials with antimicrobial surface properties as well as with enhanced mechanical and tribological properties, suitable for multiple biomedical applications. *Mahat and co-workers* discussed conducting polymers for antiviral and antimicrobial properties for PPE, such as gloves, face mask, face shield, and coverall suit for frontline health workers, to ward off bacterial infections in hospital settings, specifically in cases involving COVID-19 patients. *El-Kadi and co-workers* explained potential use of drug-loaded nanoparticles for targeting arachidonic acid–related metabolites in COVID-19 infected patients as a novel treatment strategy. *Rezaei and co-workers* summarized functional biomaterials and types of drug delivering nanoparticles for targeting nervous system, a major site that SARS-COV-2 affects.

Finally, the issue presents several papers on other novel material science applications relevant to the pandemic such as new medical devices, tissue engineering, and additive manufacturing. Mechanical ventilators are widely used in intensive care units for treating COVID-19 patients. These invasive therapies sometimes further injure infected lungs. *Chowdhury and co-workers* explored different materials to enclose the patient for negative pressure ventilators that are less invasive and suitable for pandemic situations. *Ustundag and co-workers* summarized major organs that are affected from SARS-COV-2 and different tissue engineering approaches to mitigate the effects of virulence. *Huri and co-workers* presented novel additive manufacturing applications for prevention of spread of the virus such as production of face masks, face shields, snorkels, and production of medical device parts in high demand with this approach. *Gunduz and co-workers* explained basics of 3D printing such as utilized materials and different methods of 3D printing and then explained most common 3D Printing applications relevant to COVID-19 pandemic such as printing PPEs, swabs, and even whole isolation wards.

In each published article of this special issue, there is a novel contribution of production and utilization of biomaterials for biomedical applications relevant to COVID-19. Our sincere thanks also go to the associated reviewers and editors for their unconditional help in bringing this highly informative and competitive issue to fruition. We strongly believe that this issue will serve as a best platform of knowledge transfer and help the multidisciplinary community to identify a key direction for the science and technology towards advanced emergent materials applications to fight the SARS-COV-2 and relevant complications.

